# Validating an Electronic Health Record Algorithm for Diabetes Screening Eligibility in the Emergency Department

**DOI:** 10.5811/westjem.20548

**Published:** 2025-02-13

**Authors:** Mary H. Smart, Janet Y. Lin, Brian T. Layden, Yuval Eisenberg, Kirstie K. Danielson, Ruth Pobee, Chuxian Tang, Brett Rydzon, Anjana Bairavi Maheswaran, A. Simon Pickard, Lisa K. Sharp, Angela Kong

**Affiliations:** * University of Illinois Chicago, College of Pharmacy, Department of Pharmacy Systems, Outcomes and Policy, Chicago, Illinois; † University of Illinois Chicago, College of Medicine, Department of Emergency Medicine, Chicago, Illinois; ‡ University of Illinois Chicago, Department of Medicine, Division of Endocrinology, Diabetes and Metabolism, Chicago, Illinois; § Jesse Brown Department of Veterans Affairs Medical Center, Chicago, Illinois; || University of Illinois Chicago, College of Nursing, Department of Biobehavioral Nursing Science, Chicago, Illinois

## Abstract

**Objective:**

While the American Diabetes Association (ADA) screening guidelines have been used widely, the way they are implemented and adapted to a particular setting can impact their practical application and usage. Our primary objective was to validate a best practice advisory (BPA) screening algorithm informed by the ADA guidelines to identify patients eligible for hemoglobin a1c (HbA1c) testing in the emergency department (ED).

**Methods:**

This cross-sectional study included adults presenting to a large urban medical center’s ED in May 2021. We used sensitivity, specificity, likelihood ratios, and predictive values to estimate the algorithm’s ability to correctly identify patients eligible for diabetes screening, with manual chart review as the reference standard. Eligibility criteria targeted patients at risk for diabetes who were likely unaware of their elevated HbA1c. We also calculated the area under the receiver operating characteristic curve (AUC).

**Results:**

In May 2021, 2,963 (77%) of the 3,850 adults admitted to the ED had a routine lab ordered. Among those, 796 (27%) had a BPA triggered, and of those 631 (79%) had an HbA1c test completed. The algorithm had acceptable sensitivity (0.69, 95% confidence interval [CI] 0.66–0.72), specificity (0.91, CI 0.89–0.92), positive predictive value (0.75, CI 0.72–0.78) and negative predictive value (0.88, CI 0.86–0.89). The positive likelihood ratio (7.39, CI 6.35–8.42) was adequate, and the negative likelihood ratio (0.34, CI 0.30–0.37) was informative. The AUC of 0.74 (CI 0.72–0.77) suggests that the algorithm had acceptable accuracy.

**Conclusion:**

Findings suggest that an electronic health record-based algorithm informed by the ADA guidelines is a valid tool for identifying patients presenting to the ED who are eligible for HbA1c testing and may be unaware of having prediabetes or diabetes. The ease of workflow integration and high yield of potentially undiagnosed diabetes and prediabetes makes the BPA algorithm an appealing method for diabetes screening within the ED.

## INTRODUCTION

Diabetes affects approximately 38 million adults and is the seventh leading cause of death in the United States.^1^ Type 2 diabetes accounts for over 90% of all people with diabetes.^2^ Approximately 23% of individuals with type 2 diabetes, and 80% of those with prediabetes (an intermediate high-risk condition for type 2 diabetes) are unaware of their condition.^1^ While diabetes screening has commonly been offered in primary care settings,^3^,^4^ certain populations do not routinely use primary care services.^5^–^9^ These populations include those with less educational attainment,^10^ low socioeconomic status,^11^,^12^ no or inconsistent health insurance status,^13^–^15^ certain racial and ethnic minority backgrounds,^16^–^18^ and those with poor English literacy.^19^,^20^ These groups are also more disproportionately burdened by diabetes and its associated complications.^21^,^22^ Therefore, individuals at greater risk for diabetes are less likely to be screened for diabetes in primary care.^23^

Concurrently, those disproportionately affected by diabetes frequently use emergency department (ED) services.^24^–^28^ The ED setting, traditionally thought to focus on providing emergent and urgent healthcare, may be an opportune environment to introduce targeted preventative services for populations that do not routinely use primary care services.^29^,^30^ Studies in the US have shown that diabetes screening in the ED using hemoglobin A1c (HbA1c) as the diagnostic test is feasible.^31^–^36^ Additionally, retrospective studies in the US have reported a higher prevalence of undiagnosed diabetes and prediabetes in the ED compared to the national average.^37^,^38^ The higher prevalence of undiagnosed diabetes and prediabetes in the ED may reflect the prevalence of undiagnosed disease among vulnerable populations that frequently use the ED and are at greater risk for diabetes-related complications.^23^,^24^ Therefore, offering diabetes screening and conducting HbA1c testing in the ED may provide a “safety net” to patients who underuse or have limited access to primary care services to prevent downstream complications and healthcare costs.^29^,^30^

Previously reported diabetes screening initiatives within the ED were conducted within a controlled research setting and do not reflect implementation efforts within a real-world ED practice setting.^31^–^33^ Electronic health record (EHR)-based clinical decision support tools, such as a best practice advisory (BPA), have been used for screening patients in the ED and could be an efficient tool for identifying diabetes at-risk patients in the acute care setting.^39^,^40^ A BPA is a pop-up message built into the EHR that can remind, guide, or prompt clinician action in the course of patient care.^41^,^42^ Such automated EHR-driven tools have been used successfully for identifying and screening high-risk patients for HIV^43^,^44^ and hepatitis C^45^,^46^ in the ED. In 2020, the Innovating Diabetes Screening in Emergency Departments and Linkage Services (IDEAL) project implemented a routine, EHR-based diabetes screening BPA in the ED at the University of Illinois Hospital and Health Sciences (UI Health) System. The program was designed to identify ED patients with undiagnosed prediabetes or diabetes and facilitate appropriate linkage to care, which has been described elsewhere.^47^ The BPA algorithm is informed by the American Diabetes Association (ADA) screening guidelines.^48^

**Population Health Research Capsule**

What do we already know about this issue?

*Diabetes screening in the emergency department (ED) offers diabetes prevention opportunities to patients of low socioeconomic status who may be overlooked in primary care*

What was the research question?

*Can an algorithm embedded into an electronic health record system validly screen patients at risk for diabetes in the ED?*

What was the major finding of the study?

*The algorithm is valid based on performance characteristics (eg, sensitivity, specificity, area under the ROC curve).*

How does this improve population health?

*The ease of workflow integration and high yield of potentially undiagnosed diabetes and prediabetes make the algorithm an appealing method for diabetes screening within the ED.*

While the ADA guidelines have informed previously reported diabetes screening efforts within the ED setting,^31^–^33^,^38^,^49^ the way the guidelines are implemented and adapted to a particular setting can have an important impact on their practical application or utilization.^50^–^52^ For example, using personnel to identify patients at risk for diabetes can be resource-intensive, difficult to implement within non-research settings, and prone to human error.^31^,^53^,^54^ The advent of the EHR and its clinical decision support tools, like BPAs, make it possible to use technology to aid screening initiatives and eliminate inefficiencies.^55^–^58^ While the use of clinical decision tools to identify at-risk patients is more common, only some tools have been validated to demonstrate that they are accurate in identifying the intended population.^55^,^59^ The validation of an EHR-based algorithm informed by the ADA guidelines to identify ED patients with potentially undiagnosed prediabetes or diabetes has not been previously reported. Therefore, our objective was to validate an EHR-based diabetes screening BPA informed by the ADA guidelines in identifying eligible patients for HbA1c testing in the ED using commonly extractable elements from patients’ health records.

## METHODS

### Study Population

We performed a cross-sectional cohort study of adults (≥18 years old) presenting to the UI Health ED in May 2021 with a routine diagnostic lab ordered. An automatic report generated through the hospital’s EHR (Epic Systems Corporation, Verona, WI) identified the cohort. The University of Illinois Chicago Institutional Review Board reviewed and approved this study. A visual representation of the program can be seen in the Appendix figure.

The screening algorithm was informed by the ADA guidelines and was simplified for ease of implementation.^48^ The algorithm identified those eligible for HbA1c testing based on the following criteria: 1) patients ≥45 years old; or 2) patients ≥18 years old with a body mass index (BMI) of ≥25 kg/m^2^; and 3) no history of diabetes; and 4) no HbA1c test result three years preceding the ED visit. If BMI was missing at the visit, the algorithm used the most recent BMI on file. The algorithm did not trigger a BPA if no previous BMI was available or if age was missing. The algorithm used diagnosis-related group codes associated with diabetes to identify a history of diabetes within specific searchable fields. The algorithm searched for a previous HbA1c test result in the patient’s lab results in the EHR. The algorithm could not search through free text or information shared by other health systems via the integrated EHR network (ie, EpicCare Everywhere). If a patient presenting to the ED with a routine diagnostic lab ordered met the screening criteria, the algorithm triggered a BPA, which would notify the clinician that the patient could be at risk for diabetes and was eligible for diabetes screening (ie, HbA1c testing). The clinician could then “add” a preselected HbA1c test to the existing lab order.

### Measures

The following parameters informed the EHR algorithm: age (continuous); BMI (continuous); history of diabetes (dichotomous); and history of HbA1c test within three years (dichotomous) at the time of the ED visit. The inputs for the algorithm were obtained using automatic reports generated via the EHR and manual chart extraction. Automatic reports for algorithm inputs were available for patients with a BPA triggered. No report for “potential inputs” was available for patients who did not have a BPA triggered. In the interest of time, researchers randomly sampled 10% of all patients without a BPA triggered, stratified by race and ethnicity, and identified the inputs that likely informed the BPA during the ED visit via manual chart review. Five individuals (MS, PP, JP, AA, PK) conducted a manual chart review from September 2021–October 2022 to identify reference values for checking against the algorithm inputs to verify whether the BPA was triggered or failed to be triggered appropriately. For all manual data extraction, one individual initially extracted the data, and a second individual double-checked each item.

Personnel manually verified the algorithm input for BMI against the BMI taken during the ED visit and the algorithm input for age reported against the date of birth entered in the patient’s EHR. A history of diabetes was assessed by searching for the words “diabetes,” “DM,” “type 2 diabetes,” “T2D,” “T2DM,” “preDM,” and “prediabetes” using the chart search function in Epic, which searches through notes, entries, and scanned documents for the keywords (including their synonyms) within the EHR and across the Care Everywhere network. Similarly, HbA1c history was assessed using keywords such as “HbA1c” and “hemoglobin a1c” to help parse through laboratory results. Hemaglobin A1c testing is unreliable for diagnosing prediabetes or diabetes in patients with sickle cell anemia or HIV, or in women who are pregnant.^48^ To maximize the ease of implementation, no associated exclusion criteria was built into the algorithm at the time. During the chart review, BPA triggers among patients who were identified with the aforementioned conditions during their ED visit were flagged and counted against the algorithm since further assessment is needed to diagnose these patient populations (ie, false positives).

### Statistical Analysis

We used the Student *t*-test (and Wilcoxon rank sum test for non-normally distributed data) and chi-square tests to compare continuous and categorical demographic characteristics, respectively, for the BPA-triggered and no BPA-triggered groups. We generated two-by-two tables for BPA alerts against manual chart review results. We reported estimates for sensitivity, specificity, predictive values, and likelihood ratios. The sensitivity and specificity were considered acceptable if the sum of the two values was at least 150%.^60^,^61^ The sensitivity is the algorithm’s ability to correctly identify the proportion of patients who are truly eligible for HbA1c testing in the ED. Specificity is the algorithm’s ability to identify the proportion of ED patients who are truly ineligible for HbA1c testing.^60^ The positive predictive value (PPV) represents the probability of an individual who had a BPA triggered being truly eligible for HbA1c testing, while the negative predictive value (NPV) represents the probability of an individual who did not have a BPA triggered being truly ineligible for HbA1c testing.^61^

Based on existing literature, the prevalence of undiagnosed diabetes in the ED is approximately 30%.^37^,^62^ Assuming a minimum sensitivity and specificity of 75% for both with a 30% prevalence, the minimum desired PPV and NPV are calculated to be 56% and 88%, respectively.^61^ Likelihood ratios are not influenced by disease prevalence and summarize the extent to which the algorithm changes the initial likelihood of the patient’s eligibility for HbA1c testing (ie, pretest probability) to a more accurate estimate of the patient’s eligibility (ie, posttest probability).^63^,^64^ A positive likelihood ratio (LR+) between 5–10 and a negative likelihood ratio (LR−) between 0.1–0.2 were deemed acceptable.^64^ We reported associated standard errors and 95% confidence intervals (CI).

We built receiver operating characteristic (ROC) curves to evaluate the algorithm’s accuracy using age, BMI, history of diabetes, and history of HbA1c test within three years of the ED visit based on a weighted logistic regression model. The binary outcome of interest was correct BPA determination of HbA1c eligibility, defined as the BPA firing (or not firing) appropriately when verified by the medical chart review as the reference standard. We estimated sampling weights based on the underlying distribution of race and ethnicity within the overall population. The area under the curve (AUC) was generated for each model.^65^ An AUC between 0.7–0.8 is acceptable, and above 0.8 is considered excellent.^66^ We performed all statistical analyses using SAS software version 15.2 (SAS Institute Inc., Cary, NC). Statistical analysis was completed in April 2023.

## RESULTS

In May 2021, 2,963 (77%) of the 3,850 adults presenting to the UI Health ED had a laboratory test ordered. Of those, 796 (27%) had a BPA triggered, with 631 (79%) of those triggers leading to completed HbA1c tests. A cohort of 221 patients was randomly selected for manual data extraction among the 2,167 patients who did not trigger a BPA. A cascade diagram of the algorithm can be seen in the Figure. A greater proportion of males had a BPA triggered compared to the proportion of females (33% vs 23%, *P*<0.01) (Table 1).

The BPA-triggered group tended to be older (51 vs 46, *P*<0.01) with a similar BMI (30 vs 30, *P* = 0.124) compared to the no-BPA-triggered group. Most patients across both groups were non-Hispanic Blacks, although a smaller proportion of non-Hispanic Blacks (24%) and Hispanics (29%) had a BPA triggered compared to other identified racial groups. There was a greater proportion of uninsured (39%), other (39%), and privately insured patients (33%) compared to those with Medicare (25%) and Medicaid (23%). A two-by-two table can be seen in Table 2.

The algorithm had an acceptable sensitivity (0.69, 95% CI 0.66–0.72), specificity (0.91, CI 0.89–0.92), PPV (0.75, CI 0.72–0.78) and NPV (0.88, CI:0.86–0.89) (Table 3). The LR+ was also acceptable (7.39, CI 6.35–8.42), and although the LR− (0.34, CI 0.30–0.37) was greater than the predetermined cut-off, values between 0.2 and 0.5 can still be significant in driving change in pretest to posttest probability.^64^ The AUC of 0.74 (CI 0.72–0.77) suggests that the algorithm displayed acceptable accuracy.^66^

## DISCUSSION

We sought to evaluate the accuracy of adapting the ADA guidelines to identify ED patients at risk of diabetes who were likely unaware of their condition via an EHR-based algorithm. To our knowledge, this is the first study validating a BPA diabetes screening algorithm informed by the ADA guidelines to identify eligible patients for HbA1c testing within the ED. The performance characteristics of the algorithm were acceptable, especially given the ease with which screening was integrated into the existing ED workflow and the yield of patients identified with abnormal HbA1c who would require resources to facilitate linkage to primary care. We conclude that the EHR-based algorithm informed by the ADA is a valid tool to identify patients with potentially undiagnosed prediabetes and diabetes within the ED.

The algorithm had high specificity (0.91), indicating that it was effective in correctly excluding patients who were not eligible for HbA1c testing, which is desirable when resources are limited.^67^ The high yield of abnormal HbA1c among those screened (Figure) who will require follow-up and possible linkage to care makes a highly specific screening algorithm desirable.^61^,^68^ The high NPV (0.88) minimizes the likelihood of missing eligible patients for testing among those who did not have a BPA triggered.^68^ This is important since screening in the ED provides a safety net for patients who may not be screened in other settings (ie, primary care).^29^ The PPV (0.75) is acceptable while moderate, given that the HbA1c lab draw is easily added due to the BPA and is relatively inexpensive to run.^68^,^69^

Also, the LR+ and LR− are intuitively related to ruling out and ruling in, respectively, of patient’s true eligibility for diabetes screening in the ED.^64^,^70^ The LR+ (7.4) gives moderate assurance that when the BPA did not trigger, the patient was truly ineligible for HbA1c testing. The LR− (0.34) may mean that when the BPA triggers, the patient may actually not have been eligible for testing. This likely reflects the algorithm’s inability to identify accurate identify certain inputs, which results in the BPA misfiring (eg, a patient with a history of diabetes or previous HbA1c test was not searchable in the UI Health EHR). However, overall, the algorithm displayed acceptable accuracy, as seen in the AUC value of 0.74,^66^ especially when considering that the screening implementation method (ie, EHR-based BPA) allowed for easy integration into the existing ED workflow, the cost of testing is relatively inexpensive, and the volume of patients identified with abnormal HbA1c that require care coordination follow-up.^69^

Previously reported diabetes screening programs in the ED within the US are resource-intensive and can be burdensome to implement, particularly if screening procedures disrupt the ED workflow.^31^–^33^,^53^ Screening strategies that are easily integrated into existing system processes will be essential for successful program implementation and maintenance across ED health systems.^71^,^72^ An EHR-based algorithm facilitates the ease of ED workflow integration, given that most hospitals in the US use an electronic record system and are familiar with BPAs.^73^,^74^ Previous EHR-based screening programs have been successfully implemented in acute care settings (ie, such as those for HIV and hepatitis C).^44^,^56^,^75^ Our findings and other reports from the IDEAL program affirm the feasibility and efficiency of using a routine EHR-based diabetes screening algorithm and a clinical decision support tool (such as the BPA) in the emergency care setting.^47^

The IDEAL program has found that approximately half of those tested had an abnormal HbA1c, and 75% of patients with abnormal results who were successfully followed up indicated they were unaware of their condition.^47^ This suggests that patients eligible for HbA1c testing at the UI Health ED have a significant likelihood of having an abnormal HbA1c result, likely indicative of an undiagnosed condition. These findings are consistent with previously published literature and highlight the impact this EHR-based diabetes screening tool may have.^37^,^47^,^62^

## LIMITATIONS

There are several limitations to the study. The algorithm cannot search free text (eg, clinician notes) or information shared via Epic Care Everywhere, which is a literature-reported limitations and source of misclassification errors in EHR data.^76^ Additionally, the algorithm was developed using the 2020 ADA guidelines,^48^ and our findings do not directly reflect the updated 2022 ADA guidelines, which lowered the screening age to 35.^77^ However, recent literature has found that the ADA guidelines maintained high sensitivity even when lowering the age threshold for screening.^78^ To ease the implementation process, the algorithm did not differentiate based on race (eg, Asian American), comorbidities (eg, hypercholesterolemia or hypertension), or family history (eg, first-degree relative with diabetes) since these parameters are often unreliably entered into the records but are recommended factors for consideration in the guidelines.^48^,^79^ Additionally, the algorithm did not exclude patients with a history of sickle-cell anemia, HIV, or women who were pregnant; however, BPA firings among these patients counted against the algorithm as predetermined by the research team, since HbA1c testing is unreliable in diagnosing prediabetes and diabetes in these patients.^48^ Future versions may attempt to account for these specifications. While we could not measure the true prevalence of HbA1c testing eligibility, we used previously reported estimates of undiagnosed diabetes in the ED to inform our acceptable ranges for PPV and NPV, which are influenced by disease prevalence.

Our present validation study was conducted at a single site within a one-month period. Upon expansion of the program, future validation efforts should include multiple sites across a wider timeframe. Finally, the UI Health care coordination team attempted to follow up with patients with an abnormal HbA1c result and found that approximately 25% of those who were followed up by care coordination services indicated they were previously aware of an existing prediabetes or diabetes condition. However, we could not verify this information during the chart review and possibly misclassified these patients due to missing EHR information.

## CONCLUSION

Findings suggest that an electronic health record-based algorithm informed by the American Diabetes Association guidelines is a valid tool for identifying patients presenting to the ED who were at risk for diabetes and, if upon testing had an elevated HbA1c, were likely unaware of having prediabetes or diabetes. The ease of workflow integration and high yield of potentially undiagnosed diabetes and prediabetes make the BPA algorithm an appealing method for diabetes screening within the acute care setting. Future research initiatives include validating any updates to the algorithm and exploring effective linkage to care strategies and cost-effectiveness studies.

## Supplementary Information



## Figures and Tables

**Figure:**
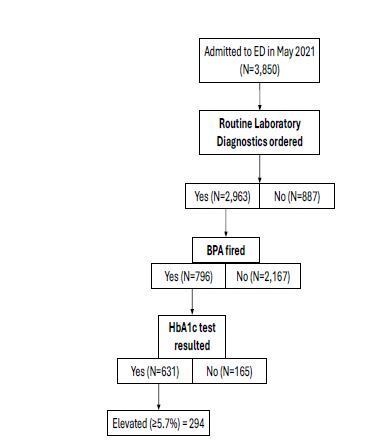
Cascade diagram of patients eligible for diabetes screening in the emergency department. *ADA*, American Diabetes Association; *BPA*, best practice advisory; *ED*, emergency department.

**Table 1:** Patients with best practice advisory triggered vs none triggered based on the modified American Diabetes Assocation screening algorithm.

	Total cohort (N=2,963)	BPA triggered^a^ (n=796)	No BPA triggered (n=2,167)	P-value^b^
Sex, n (%)				<0.01
Male	1,182 (39.9)	394 (33.3)	788 (66.7)	
Female	1,781 (60.1)	402 (22.6)	1,379 (77.4)	
Age, mean (SD)	48 (17.9)	51 (17.1)	46 (18.1)	<0.01
Age, n (%)				<0.01
18–44, n (%)	1,376 (46.4)	279 (20.3)	1,097 (79.7)	
45–64, n (%)	1,003 (33.9)	330 (32.9)	673 (67.1)	
65+, n (%)	584 (19.7)	187 (32)	397 (68)	
BMI, mean (SD)	30.1 (8.8)	30.4 (8.7)	30 (8.9)	0.24
Race/ethnicity, n (%)				<0.01
Hispanic	763 (25.8)	218 (28.6)	545 (71.4)	
White, non-Hispanic	290 (9.8)	114 (39.3)	176 (60.7)	
Black, non-Hispanic	1,649 (55.7)	389 (23.6)	1,260 (76.4)	
Asian, non-Hispanic	99 (3.3)	38 (38.4)	61 (61.6)	
Other, non-Hispanic	125 (4.2)	29 (23.2)	96 (76.8)	
Unknown	37 (1.3)	8 (21.6)	29 (78.4)	
Insurance, n (%)				<0.01
Medicaid	1,374 (46.4)	316 (23)	1,058 (77)	
Medicare	760 (25.7)	192 (25.3)	568 (74.7)	
Private	613 (20.7)	204 (33.3)	409 (66.7)	
Other	36 (1.2)	14 (38.9)	23 (63.9)	
Uninsured	180 (6.1)	70 (38.9)	110 (61.1)	

a Informed by the American Diabetes Association 2020 screening guidelines, focused on patients 1) ≥18 with a BMI ≥ 25 kg/m2 or ≥ 45 years old; and 2) without a previous diabetes diagnosis and without a previous hemoglobin A1c test in their chart within the previous 3 years.^48^

b Boldface indicates statistical significance (P<0.05).

*BMI*, body mass index; *BPA*, best practice alert.

**Table 2:** Modified American Diabetes Assocation- and simulated US Preventive Services Task Force-electronic health record algorithm best practice alerts by manual chart review.^a^

EHR algorithm	ADA Manual chart review		

	The BPA should have triggered	The BPA should not have triggered	Total
BPA triggered	599	197	796
No BPA triggered	265	1,902	2,167
Total	864	2,099	2,963

a The upper left-hand quadrant represented true positives (ie, patients for whom BPA triggering was deemed appropriate after verifying the four parameters of interest through the manual chart review process). The lower right-hand quadrant represented true negative (ie, patients for whom BPA correctly failed to trigger, also verified by manual chart review). The upper right-hand quadrant represented false positives (ie, patients for whom a BPA was triggered when it should not have been based on manual chart review. Finally, the lower left-hand quadrant represented false ineligible patients (ie, patients for whom the BPA failed to trigger but should have based on manual chart review).

*ADA*, American Diabetes Association; *BPA*, best practice advisory; *EHR*, electronic health record.

**Table 3:** Test characteristics of modified American Diabetes Association- and simulated US Preventive Services Task Force-informed electronic health record screening algorithm.

	ADA		

	Estimate	SE	95% CI
Sensitivity	0.69	0.02	(0.66 – 0.72)
Specificity	0.91	0.01	(0.89 – 0.92)
PPV	0.75	0.02	(0.72 – 0.78)
NPV	0.88	0.01	(0.86 – 0.89)
LR+	7.39	0.53	(6.35 – 8.42)
LR−	0.34	0.02	(0.30 – 0.37)
AUC	0.74	0.01	(0.72 – 0.77)

*AUC*, area under the receiver operator curve; *CI*, confidence interval; *ED*, emergency department; *HbA1c*, hemoglobin A1c; *LR+*, positive likelihood ratio; *LR−*, negative likelihood ratio; *NPV*, negative predictive value; *PPV*, positive predictive value; *SE*, standard error.
