# Supplementary material for: Validating an Electronic Health Record Algorithm for Diabetes Screening Eligibility in the Emergency Department
Source: West J Emerg Med. 2025 Feb 13;26(3):720–8. doi: 10.5811/westjem.20548 (PMC12208037; doi:10.5811/westjem.20548)
Supplement: Supplementary file 1 [file wjem-26-720-s001.docx]

**Appendix**

**Appendix Figure 1.** The Innovating diabetes screening in emergency departments and linkage services (IDEAL) program.

**
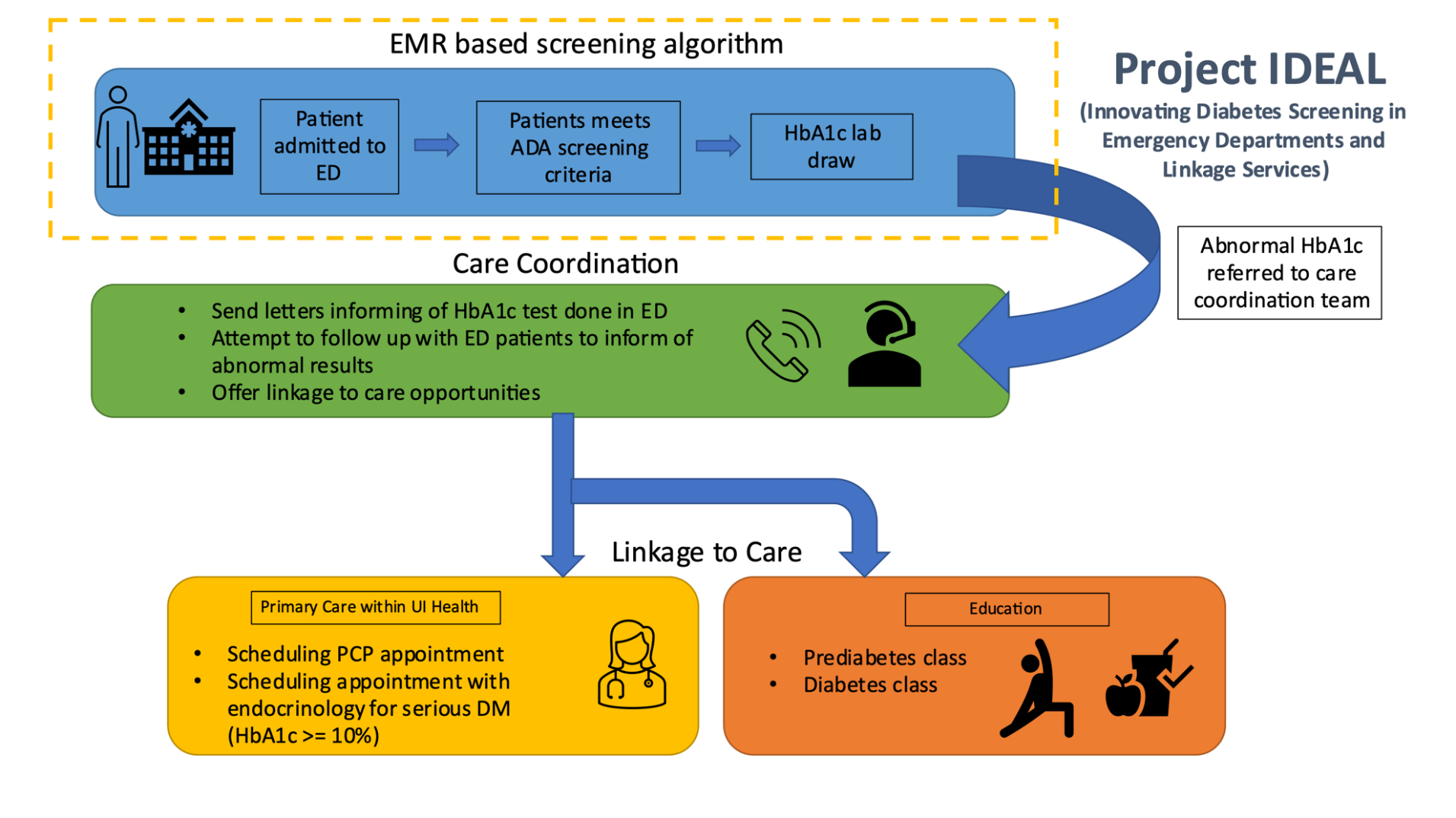
**

ED, emergency department; EMR, electronic medical record; DM: diabetes mellitus; HbA1c, hemoglobin A1; PCP: primary care provider; UI Health: University of Illinois Health System.

The yellow dashed line highlights the part of the program associated with the EMR algorithm.
